# Students learning experiences during COVID-19: Work from home period in Malaysian Higher Learning Institutions

**DOI:** 10.1177/0144739420977900

**Published:** 2020-12-06

**Authors:** Mahiswaran Selvanathan, Nur Atikah Mohamed Hussin, Noor Alyani Nor Azazi

**Affiliations:** School of Social Sciences, Universiti Sains Malaysia (USM), Malaysia

**Keywords:** Interaction, instruction, instructor, course management, technology

## Abstract

Recently, the whole globe was affected by the Coronavirus disease (COVID-19), which caused a major disruption in every economy sector as well as the education sector. Most of the education systems in the world shifted to a full online learning method, either conducted in a synchronous or asynchronous method. Thus, making the traditional teaching and learning methods were no longer option of learning method. This reality of online teaching and learning methods by the Malaysian education system, especially the Higher Learning Institution as an alternative teaching method is compulsory throughout the pandemic. This paper evaluates the experience of the students of higher learning institutions in Malaysia with the implementation of online learning during this pandemic.

## Background

The global disruption by Coronavirus disease (COVID-19 caused by a newly discovered coronavirus (World Health Organization (WHO), 2020) has severely affected the economy sector of every nation in this world, including the higher education sector. Higher education was significantly affected by this disease faced major changes that impacted the students in higher education sector. These impacts include social distancing, quarantines, isolation measures, campus closure, border closures, and travel restrictions (QS, 2020). Universities around the world were facing unprecedented challenges, including both the teaching committee and the students. In Latin America and the Caribbean, the temporary closure of higher education institutions has affected approximately 23.4 million higher education students and 1.4 million teachers in these regions that represented more than 98% of the region’s population of higher education students and teachers (UNESCO IESALC, 2020). The decision to temporarily close the higher learning institutions made by the policymakers was done due to the principle of large gatherings possessed a higher risk during this pandemic. Universities across the globe were forced to go for a lockdown and closing all the campuses had to switch the learning method towards online learning and digital tools or known as e-learning as the replacement.

### Online learning experience globally

Due to the spread of this disease and the closure of physical classes, online learning through the uses of several devices like computers, laptops, tablets and mobile phones with internet access in synchronous and asynchronous environments become the alternative learning methods. Through these learning methods and environments, students have a freedom in learning and get connected with their teachers anywhere they want (Singh and Thurman, 2019). There are two modes in online learning, which are synchronous and asynchronous depending on the application of applying optional timing of interaction (Algahtani, 2011). The synchronous online learning provides the direct interaction between the lecturers and students during class through tools such as videoconference or chatrooms. While asynchronous online learning provides the opportunity for the lecturers and students to interact before or after the online class through thread discussion and emails. The online learning provides advantages in independent learning and developing new skills in the process leading to life-long learning (Dhawan, 2020).

However, the online learning can be challenging to the disabled, underprivileged, and marginalized students who had limited resources and accessibility to online learning (The Regional Risk Communication and Community Engagement (RCCE) Working Group, 2020). This inability to access and involve in online learning causes the disparity and dropout among them. Online learning also requires students’ commitment and discipline, especially for vulnerable students who need interaction that allows them to strengthen their social skills (UNESCO IESALC, 2020).

Online learning provides advantages for both students and universities. In developed countries such as Australia and Korea, the students opted to have flexible time for learning, which is more convenient for them to access the teaching materials. The universities also gain benefits through the implementation of this learning method, which is more cost-effective with a wide audience and no necessities of physical infrastructure. (Misko et al., 2004). However, the online teaching has a few limitations as the lecturers faced difficulty in preparing materials for the online method, which is a very time-consuming process. Also, the online learning become challenging tasks in assisting the students in accessing the learning materials as online learning requires less supervision.

Online learning has emerged as a new method of learning for developing countries (Iqbal and Ahmad, 2010). There are problems that arise as online learning is introduced to the developing countries (Folorunso et al., 2006; Siritongthaworn et al., 2006). The students need to adapt themselves to the new environment computer-led training in virtual classrooms from traditional classroom, which is a challenging task (Sanchez-Gordon and Luján-Mora, 2014). Many educations institutional facing inadequate supply of e-learning equipment necessities, such as highly efficient devices and Internet connections. Some of the students have poor computer literacy and self-motivation, which affected them to access the online learning (Randy, 2011). Bhuasiri (2012) indicated the obstacles in online learning for developing countries including investments in technology such as hardware, software licenses, learning material development, equipment maintenance, and training. Besides, there are also some issues related to management support.

### Online learning experience: Malaysia case of COVID-19

The Malaysian Higher Learning Institutions had implemented online learning started in the late 1990s (Hussin et al., 2009). The online learning demand has been increasing due to the capability to reach global audiences, unique functionality, accessibility, and flexibility in the long run (Azhari and Ming, 2015). In line with the educational developments, the Malaysian Ministry of Education under the Malaysian Education Blueprint 2015–2025 (Higher Education) has introduced initiatives in making the online learning as an integral component of higher education and lifelong learning (Malaysian Education Blueprint 2015–2025 (Higher Education): E-16).

However, there are persistent concerns about the quality of online learning relative to a face-to-face learning environment (Panyajamorn et al., 2018). A study in the UK reported the students preferred face to face teaching and learning experience even they are high computer literacy (Orton-Johnson, 2009). A study in Malaysia has reported students’ computer and internet efficacy, and personal characteristics such as gender, ethnicity, course year level, and financial aid status affecting the students’ online learning readiness (Lau and Shaikh, 2012).

Nevertheless, the campus closure and movement restriction order affected the formal learning and thus, opted online learning as the best alternative to continue the learning process. In April 2020, the Malaysian government has been ordered the students to return to their hometown and continue their studies via online learning (‘Pelajar IPT’, 2020). The Malaysian government has played important roles in providing assistance by providing the internet allowance to the B40 family and students for them to access the internet and continuing their online learning (‘Internet Allowance’, 2020). This allowance allows the students to get free internet access for online learning.

Online learning is an effective alternative learning method for both of students and lecturers, however, there are some issues require consideration, such as the limited accessibility to the internet. There has reported about 52% of students in Sabah, Malaysia do not have the access to the internet due to the inadequate of infrastructures (‘52 Peratus’, 2020). Inadequate online learning infrastructures and limited accessibility to the internet make the online learning process harder for the students, (Lee, 2020) especially in more rural and isolated areas in Malaysia. Beside the limited accessibility to the internet, the students experience difficulty in communicating with their lectures, interaction with their friends, and laboratory access, which affected their studies.

The aim of this paper is to study on the students’ learning experience through online teaching methods during Covid-19 lockdown period in Malaysia. This study is vital to study the challenges experienced by Malaysian university students during the pandemic situation and provides insight to the Malaysian government in assisting the online learning at Malaysian universities.

## Methods

The target population of this research was the students from various public and private universities in Malaysia. The target population of this research was the students from various public and private universities in Malaysia. The sample size of this study is 384 respondents from 12 different public and private universities. According to Krejcie and Morgan (1970), the maximum number of sample size for large population is 384. Therefore, the number of sample size of this study is sufficient to represent the population. This study used a purposive sampling method to select the targeted respondents which are the students from public and private universities in Malaysia. The respondents also included the students that attached to any of the branches of that university. The instruments of this survey were adapted from Naaj et al. (2012). The instruments were divided into five (5) dimension which are Interaction, Instruction, Instructor, Course Management, and Technology with the Cronbach alpha of 0.75, 0.84, 0.70, 0.57, and 0.80, respectively. For the data collection, out of the 384 online survey distributed, about 370 (96.35%) responded to the survey. However, 42 survey were rejected due to being incomplete, therefore about 328 (85.41%) survey were deemed valid. Data were analysed using IBM SPSS Statistics version 25.0. Descriptive statistics were used to organize, summarize, and describe the data by mean, standard deviation, percentage, and frequency. The obtained results were used to answer the main objective of this research to investigate students’ learning experience through online teaching methods during Covid-19 lockdown period in Malaysia. From the instrument used, the data was analysed based on the mean value as follows; 1.00–2.33 is considered as low, 2.34–3.66 is considered as medium and 3.67–5.00 is considered as high. The formula for this classification is shown below (Julie, 2010):


$$\begin{array}{l} 5-1\;=\;4\\ \frac{4}{3}=1.33\\ \text{Interval}\;\text{class}=1.33 \end{array}$$

Mean Classification:


$$\begin{array}{l} \text{Low:}\;1+1.33=2.33\\ \text{Medium:}\;2.34+1.33=3.66\\ \text{High:}\;3.67+1.33=5.00 \end{array}$$

## Results

Data were collected from 328 respondents of 12 different public and private universities. Out of 328 respondents, there were 155 (47.3%) male and 173 (52.7%) female. Based on the data, the respondents of this study were 87 (26.5%) students pursuing their diploma studies, 119 (36.3%) students pursuing their degree studies, and 122 (37.2%) students pursuing their masters’ studies. 180 (54.9%) were international students and 148 (45.1%) were local students.

This result refers to the main objective of this research, to study the students learning experiences through online teaching methods during Covid-19 lockdown period in Malaysia. The Likert scale used for the customer satisfaction questionnaire was ranked from 1 to 5. The average value for the scale would be 3. The obtained mean value with above the average value of 3, showing the students had a high agreement with the items tested in this study. Table 1 demonstrates the mean values for students’ learning experiences through online teaching methods. From the results, the students showed a high dissatisfaction with the fact that they cannot interrupt the lecturers during the teaching. There are some barriers in the interaction between lecturers and students throughout online teaching. Besides, limited internet access affected the implementation of online teaching harder for the students in rural areas (‘Limited Internet’, 2020). The result also showed the students’ dissatisfaction with the group activity conducted throughout the pandemic. Some Malaysians cast doubt on the effectiveness of teaching mode using virtually due to their insufficient preparation to adopt online learning as the new method of learning throughout the pandemic.

**Table 1.** Interaction between lecturers and students when comes to online teaching.

	N	Mean
**Interaction**	328	2.8871
1 Online learning sessions keep me always alert and focused	328	3.1694
2 Interaction is adequately maintained with the lecturer	328	3.5968
3 These teaching methods make it more important for students to visit the lecturer face to face more frequently	328	3.1048
4 I cannot interrupt the lecturer to ask a question when he/she is lecturing	328	3.0081
5 I am satisfied with the quality of interaction between all involved parties	328	3.0403
6 I am dissatisfied with the process of collaboration activities during the course	328	3.1855
7 I am satisfied with the way I interact with other students	328	3.4597
8 I am satisfied with my participation	328	3.4919
**Instruction**		
1 The use of online learning technology in this course encourages me to learn independently	328	2.6532
2 My understanding is improved compared to similar courses I studied before	328	2.7823
3 My performance in exams is improved compared with similar courses I studied before	328	3.2097
4 I am satisfied with the level of effort this course required	328	3.1129
5 I am dissatisfied with my performance in this course	328	3.1613
6 I believe I will be satisfied with my final grade in the course	328	3.2984
7 I am satisfied with how I am able to apply what I have learned in this course	328	2.9032
8 I am willing to take another course or repeat the course when they are taught it in traditional delivery mode	328	3.2742
9 I am satisfied enough with this course to recommend it to others	328	3.5000
10 Compared with face-to-face course settings, I am less satisfied with this learning experience	328	3.4435
11 I enjoy working on assignments by myself	328	3.3710
**Instructor**		
1 The instructor makes me feel that I am a true member of the class		
2 I am dissatisfied with the accessibility and availability of the instructor	328	3.8629
3 The instructor uses technology appropriately	328	3.4919
4 Class assignments were clearly communicated to me	328	3.4839
5 Feedback on evaluation of tests and other assignments was given in a timely manner	328	3.4355
**Course Management**		
1 Discipline is highly observed by the lecturer	328	3.5726
2 The lecturer always takes attendance	328	3.8387
3 I attend these classes the same way I attend face-to-face classes	328	3.3387
**Technology**		
1 The instructor’s voice is audible	328	3.3629
2 Course content shown or displayed on the smart board is clear	328	3.6613
3 The microphone is in good working condition	328	3.3548
4 The video image is clear and comprehensive	328	3.4839
5 Technical problems are not frequent, and they do not adversely affect my understanding of the course	328	3.0323
6 The technology used for online teaching is reliable	328	3.3387

For the Instruction section, the result exhibited that the students showed high dissatisfaction with their performances during COVID-19. There had reported that the students preferred to take the courses in traditional delivery mode rather than online. Most of the students were dissatisfied with the implementation of virtual learning mode during this pandemic (Aiman, 2020). Besides, proper technology is a requirement to improve the internet access, so the students freely access the internet for their classes as well as for gaining their study materials. Some universities provided a limited e-library services for downloadable resources.

For the Instructor section, the students showed high disagreement with the accessibility and availability of the instructor. The lecturers faced difficulty in teaching virtually due to their insufficient preparation in adopting the new method for teaching. Due to this limitation, the students in urban areas gained benefits of the technology compared to who lived at rural areas. Limited internet access is challenging to the students in rural areas for their studies, which also resulted in delay for the task submission. According to Yeoh (2020), most students in rural schools don’t own phones and faced difficulty in e-learning during MCO. So, this result showed that Malaysia as a whole are not ready for implementing any new type of working system virtually. The limitation would have been solved to date if Malaysia started practicing this new norms since other developed countries started practicing working virtual today rather than due to the pandemic has been alerted us.

Overall, it showed that students are still coping with the new norms working from the home period, but they had dissatisfaction with certain elements that may need to be considered. The universities should take necessary action accordingly before losing their clients (students) due to this pandemic. Universities should be considering the implementation of online teaching as a new method for teaching and learning processes in the future.

## Conclusion

The discussion from the above mentioned showed that the online learning and teaching in Malaysia required improvement to be done, especially in the quality of the interaction and instruction delivered during the course given to the students. Even though the respondents are satisfied with the Instructor, course management, and also the technology dimension, some improvements should be implemented to enhance the delivery of online teaching and learning in Malaysia. Overall, the students are coping with the work from the home period, but there was dissatisfaction with certain elements. The universities should act accordingly before losing their clients (students) due to this pandemic. Especially, in Malaysian Private Higher Learning Institutions the issues are quite serious. 44 percent of Malaysian Private Higher Learning Institutions are facing financial crisis and their debt level keep increasing due to their operational cost. Parents are also suffering from this Covid-19 pandemic and every cent they are spending are counted. So, the parents will become more demanding and critical choosing the universities. They will be more calculative in the way universities are operating especially the private institutions due to the higher fee structure and the universities were chosen by the parents specially for their child’s education growth. The real issues with Malaysian Private Higher Learning Institutions, they are more depending on these international students compared to the Malaysian Public Higher Learning Institutions. So, this pandemic really hurts the enrollment of international students and obviously most international students love to come to Malaysia physically due the lovely environment, food, people and provides a good working platform after completing their studies. So, if the courses are conducted online, the students might choose any other universities or countries because their main ambition is only to get the education and not any other. This paper highlights the serious action that need to be taken by all the universities and make their courses interesting and innovative for the international students to pursue their studies.Universities should also be considering the implementation of online teaching as a new method for teaching and learning processes in the future. To highlight, Ćukušić (2010) named a few recommendations for increasing e-learning use: review current programmes, set clear goals and expected outcomes of the programme, set learning methods and activities, define the organization and presentation of activities, define learning material for every activity, select appropriate assessment models, identify skills and other requirements for access and set requirements related to resources and infrastructure.
